# The Effect of Silver Nanoparticles/Titanium Dioxide in Poly(acrylic acid-*co*-acrylamide)-Modified, Deproteinized, Natural Rubber Composites on Dye Removal

**DOI:** 10.3390/polym16010092

**Published:** 2023-12-28

**Authors:** Supharat Inphonlek, Chaiwat Ruksakulpiwat, Yupaporn Ruksakulpiwat

**Affiliations:** 1 School of Polymer Engineering, Institute of Engineering, Suranaree University of Technology, Nakhon Ratchasima 30000, Thailand; supharat.inph@gmail.com; 2 Research Center for Biocomposite Materials for Medical Industry and Agricultural and Food Industry, Suranaree University of Technology, Nakhon Ratchasima 30000, Thailand

**Keywords:** modified natural rubber, poly(acrylic acid-*co*-acrylamide), natural rubber composites, silver nanoparticle, titanium dioxide, dye removal

## Abstract

This work aims to enhance the dye-removal performance of prepared poly(acrylic acid-*co*-acrylamide)-modified, deproteinized, natural rubber ((PAA-*co*-PAM)-DPNR) through incorporation with silver nanoparticles/titanium dioxide. The (PAA-*co*-PAM)-DPNR was prepared by emulsion-graft copolymerization with a grafting efficiency of 10.20 ± 2.33 to 54.26 ± 1.55%. The composites based on (PAA-*co*-PAM)-DPNR comprising silver nanoparticles and titanium dioxide ((PAA-*co*-PAM)-DPNR/Ag-TiO_2_) were then prepared by latex compounding using the fixed concentration of AgNO_3_ (0.5 phr) and varying concentrations of TiO_2_ at 1.0, 2.5, and 5.0 phr. The formation of silver nanoparticles was obtained by heat and applied pressure. The composites had a porous morphology as they allowed water to diffuse in their structure, allowing the high specific area to interact with dye molecules. The incorporation of silver nanoparticles/titanium dioxide improved the compressive modulus from 1.015 ± 0.062 to 2.283 ± 0.043 KPa. The (PAA-*co*-PAM)-DPNR/Ag-TiO_2_ composite with 5.0 phr of TiO_2_ had a maximum adsorption capacity of 206.42 mg/g, which increased by 2.02-fold compared to (PAA-*co*-PAM)-DPNR. The behavior of dye removal was assessed with the pseudo-second-order kinetic model and Langmuir isotherm adsorption model. These composites can maintain their removal efficiency above 90% for up to five cycles. Thus, these composites could have the potential for dye-removal applications.

## 1. Introduction

Functional polymeric materials have received much attention for their potential in various applications. Since they contain functional groups, they can bind with therapeutic agents that carry them to the target in drug-delivery systems [1,2] and adsorb pollutants such as heavy metals and dyes in wastewater treatment [3,4,5]. For the removal of contaminants, functional materials have been used as templates to interact with specific molecules to remove them from a mixture. In the case of separation in aqueous conditions, functional polymeric materials should also be stable in an aqueous medium. They should maintain a dimensional structure and not deform in the medium to be reused [6]. Additionally, the templates fabricated from bio-based polymeric materials have advantages because they are environmentally friendly and have low toxicity. Among natural polymers, natural rubber (NR) is a biopolymer from *Hevea brasiliensis* rubber trees, which is interesting for the construction of bio-based devices [7,8]. NR has been extensively used in many applications because it has good mechanical properties and high elasticity. It possesses hydrophobic characteristics due to the cis-1,4-polyisoprene in its chemical structure. Therefore, the fabrication of green adsorbent materials based on natural rubber, which is a renewable biopolymer, is more suitable in terms of environmental friendliness and the ability to maintain structural stability in an aqueous environment. It can also be modified with other components to have desired functional groups for specific binding with adsorbate molecules [9,10]. 

In our previous work, deproteinized natural rubber was modified by grafting it with poly(acrylic acid-*co*-acrylamide) through emulsion-graft copolymerization in the presence of N′,N′-methylenebisacrylamide as a crosslinking agent [11]. This method is a water-based system with an environmentally friendly process. The modified natural rubber was then mixed with silica and employed as a coating material for fertilizer. After modification, the natural rubber-based material had additional functional groups, including carboxylic acid, amide, and hydroxyl groups. The presence of these functional groups not only improved water absorption capacity but also interacted with the other molecules. Maijan et al. prepared polymeric network hydrogel based on polyacrylamide and natural rubber [12]. The addition of natural rubber improved the toughness of polyacrylamide-based hydrogel and showed a higher compressive modulus compared to pure polyacrylamide hydrogel. The prepared hydrogel served as an adsorbent for dye removal that could adsorb methylene blue with a maximum adsorption capacity of 538.3 mg/g. Its removal efficiency remained at 70% after reuse for 10 cycles. 

To prepare a high-performance adsorbent, photocatalyst particles were added to improve the efficiency of the dye removal. Titanium dioxide (TiO_2_) is an inorganic compound that is used in a wide range of applications. It has been reported that TiO_2_ exhibits excellent photocatalytic activity [13,14]. TiO_2_ can produce reactive oxygen radical species under UV irradiation. The radicals can degrade organic pollutants in wastewater into harmless products. However, there are some drawbacks; for example, the use of TiO_2_ powder for treatment may not be recyclable, and it can only absorb UV light (band gap 3.2 eV). Many research works have reported the use of TiO_2_ incorporated into metals to improve efficiency in visible regions. Suriyachai et al. prepared modified TiO_2_ particles for photocatalytic glucose conversion [15]. The TiO_2_ was modified by doping with metal or metalloid (B and Ag) and nonmetal (N). The increase in absorbance in the visible region was found with the single doping (Ag) and co-doping (Ag/N and B/N). The use of Ag-doped TiO_2_ enhanced the glucose conversion to 63.5%, while the glucose conversion of bare TiO_2_ was only 24.9%. The conversion was further improved with co-doping (>90%). Therefore, the modified TiO_2_ could improve performance in terms of productivity. Generally, metallic particles can be formed by the reduction of metal ions. However, strong reducing agents are often added in this method that may be harmful. Wongpreecha et al. prepared silver nanoparticles through a one-step green process [16]. The silver nanoparticles were generated under high temperature and pressure using AgNO_3_ as a metal precursor and chitosan as a reducing agent and stabilizer. The synthesized silver nanoparticles exhibited good long-term stability and antibacterial activity. In addition, it has been reported that polyacrylic acid can act as a host material for silver nanoparticles since the carboxylic groups in its structure can form coordination or ionic bonds with silver ions [17,18]. Fahmy et al. prepared a thin film of polyacrylic acid incorporated into silver nanoparticles by plasma polymerization [19]. The aluminum substrates covered with polyacrylic acid were saturated with Ag^+^ ions (from an AgNO_3_ solution). The reduction process to generate silver nanoparticles was performed by two methods, which were (1) the use of NaBH_4_ as a reducing agent and (2) sunlight exposure. The backbone structure of the polymer layer was not changed by exposure to sunlight. Silver nanoparticles with a size of less than 5 nm were formed and homogeneously dispersed in the polymer layer. 

In this work, poly(acrylic acid-*co*-acrylamide)-modified, deproteinized, natural rubber was prepared via emulsion-graft copolymerization and used as a polymer matrix to fabricate an adsorbent material for dye removal. Various factors in polymerization were then studied, including crosslinking agent contents, ratio of comonomer, and monomer contents. Novel composite materials were prepared by incorporating silver nanoparticles and titanium dioxide into the modified natural rubber through a simple process to enhance the efficiency of dye removal. The method to obtain silver nanoparticles dispersed in the polymer matrix was performed under heat and pressure without the addition of any harmful reducing agents. The properties of the obtained composites were characterized, and the ability of dye removal was investigated. 

## 2. Materials and Methods

### 2.1. Materials

Natural rubber latex (NR; 60% of dry rubber content preserved with high ammonia) was obtained from Chemical and Materials Co., Ltd. (Bangkok, Thailand). The proteins were removed from NR latex using urea and sodium dodecyl sulfate, following the method by Kawahara et al. [20], to obtain deproteinized natural rubber (DPNR) latex. Urea was received from RCI Labscan Limited (Bangkok, Thailand). Sodium dodecyl sulfate and acrylamide (AM) monomer were purchased from Loba Chemie Pvt. Ltd. (Mumbai, India). Acrylic acid (AA) and cumene hydroperoxide (CHP) were obtained from Aldrich (St. Louis, MO, USA). The AA monomer was purified by passing through a column packed with alumina adsorbent before use [21]. Tetraethylene pentamine (TEPA) was purchased from Acros Organics (Geel, Belgium). Terric16A (10 wt.%) was obtained from the Rubber Authority of Thailand (Bangkok, Thailand). N′,N′-Methylenebisacrylamide (MBA) was received from Alfa Aesar (Haverhill, MA, USA). Silver nitrate (AgNO_3_) was obtained from Quality Reagent Chemical (Controlled by QReC New Zealand; Rawang, Malaysia). Titanium dioxide (TiO_2_; anatase, a residue from a 45 µm sieve ≤ 0.1% and oil absorption value of 26 g/100 g) was received from Cernic International Co., Ltd. (Nakhon Pathom, Thailand). Methylene blue (MB) was obtained from KEMAUS (New South Wales, Australia). Deionized (DI) water was used throughout the study. 

### 2.2. Preparation of Poly(acrylic acid-co-acrylamide)-Modified, Deproteinized, Natural Rubber

The procedure for preparing the poly(acrylic acid-*co*-acrylamide)-modified, deproteinized, natural rubber ((PAA-*co*-PAM)-DPNR) was carried out via emulsion-graft copolymerization of the comonomers of acrylic acid and acrylamide, according to previous work [11]. The DPNR latex was mixed with Terric16A using a mechanical stirrer at 100 rpm in a three-necked round-bottom reactor. The dispersion was purged with nitrogen gas for 45 min. Subsequently, CHP, acrylic acid, acrylamide, MBA, and TEPA were injected into the reactor. Acrylic acid (50 mol%) was neutralized with a 20 wt.% of NaOH solution. CHP and TEPA were fixed at 1 phr. The total solid content was adjusted to 20 wt.% with deionized water. Polymerization parameters were set at 50 °C for 6 h under a nitrogen atmosphere. Finally, the (PAA-*co*-PAM)-modified DPNR latex was formed. 

### 2.3. Characterization of (PAA-co-PAM)-Modified, Deproteinized, Natural Rubber 

#### 2.3.1. Determination of Monomer Conversion, Grafting Efficiency, and Grafting Percentage

The obtained latex was dried in a hot-air oven at 60 °C. The dried samples were weighed and then immersed in ethanol for 24 h to eliminate any unreacted monomers. Subsequently, the samples were dried again in a hot-air oven at 60 °C for 24 h. The monomer conversion was calculated using the following equation [22]:

(1) $$Monomer\;conversion\left(\%\right)=\frac{Weight\;of\left(PAA\text{-}co\text{-}PAM\right)\;formed}{Weight\;of\;total\;monomer\;added}\times 100.$$

To determine the grafting efficiency and grafting percentage, the extraction of samples with DI water was conducted to separate the ungrafted PAA-*co*-PAM. The samples were immersed in DI water for 72 h, and the DI water was changed three times a day. The samples were subsequently dried at 60 °C overnight. The weights of the samples before and after extraction were recorded. The grafting efficiency and grafting percentage were calculated as follows [23]:

(2) $$Grafting\;efficiency\left(\%\right)=\frac{Weight\;of\left(PAA\text{-}co\text{-}PAM\right)\;grafted}{Weight\;of\;total\;polymer\;formed}\times 100$$

(3) $$Grafting\;percentage\left(\%\right)=\frac{Weight\;of\left(PAA\text{-}co\text{-}PAM\right)\;grafted}{Weight\;of\;DPNR\;used}\times 100.$$

#### 2.3.2. FTIR Analysis

Fourier transform infrared spectroscopy (FTIR) was employed to characterize the chemical functional groups of the prepared samples. The measurements were performed at a resolution of 4 cm^−1^ with 64 scans using attenuated total reflection (ATR) mode on an FTIR spectrometer (Tensor 27, Bruker, Billerica, MA, USA). The scanning was recorded in the range of 4000 to 500 cm^−1^. 

### 2.4. Preparation of (PAA-co-PAM)-DPNR/Ag-TiO_2_ Composites

**Figure 1:**
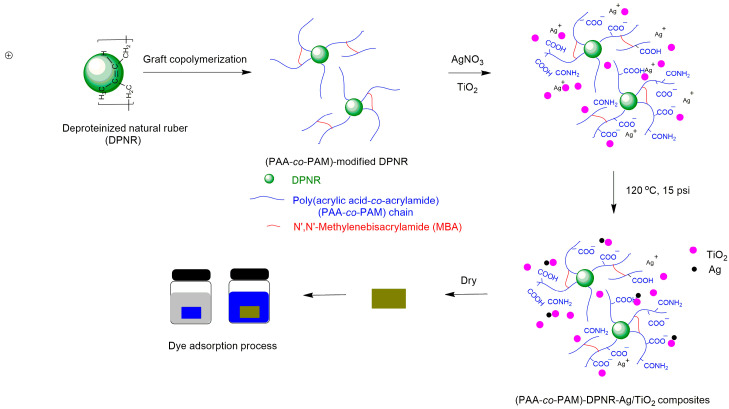
Schematic illustration of the preparation of the (PAA-*co*-PAM)-DPNR/Ag-TiO_2_ composites for the adsorption of dye molecules.

The (PAA-*co*-PAM)-DPNR composites consisting of silver nanoparticles and titanium dioxide were prepared and utilized as adsorbent materials, as shown in Figure 1. The composites were prepared by mixing the (PAA-*co*-PAM)-modified DPNR latex with a 1% *w*/*v* AgNO_3_ solution and a 10% *w*/*v* TiO_2_ dispersion. The concentration of AgNO_3_ was fixed at 0.5 phr, while the TiO_2_ contents varied at 1.0, 2.5, and 5.0 phr. The mixture was magnetically stirred at 600 rpm for 30 min under dark conditions. Subsequently, the mixture was transferred to an autoclave (HVA-110, Hirayama Manufacturing Corporation, Saitama, Japan). The experiment was conducted at 120 °C under pressure of 15 psi for 50 min. The obtained latex was poured into a silicone mold and dried in a hot-air oven at 60 °C for 24 h. The samples were purified by extraction with DI water before use. Control samples loading either AgNO_3_ or TiO_2_ alone were also prepared. 

### 2.5. Characterization of (PAA-co-PAM)-DPNR/Ag-TiO_2_ Composites

#### 2.5.1. UV-Vis Spectroscopy

The formation of silver nanoparticles was investigated using a UV-vis spectrophotometer (Cary Series, Agilent Technologies, Santa Clara, CA, USA). The diluted latex loaded with AgNO_3,_ both before and after autoclaving, was added into the cuvette and subjected to the sample holder. Latex without the addition of AgNO_3_ was used as a blank. The measurement was conducted from 300 to 800 nm at a scan rate of 600 nm/min.

#### 2.5.2. X-ray Diffraction

The crystal structure of silver nanoparticles was investigated using X-ray diffraction (XRD, D2 PHASER, Bruker, Billerica, MA, USA) with a Cu Kα radiation source operated at 30 kV and a current of 10 mA. The XRD patterns were recorded over the 2θ range of 30° to 80° with a scan rate of 2°/min and a step size of 0.02°.

#### 2.5.3. Morphology 

The morphology of different types of composites was observed by scanning electron microscopy (SEM). The dried samples were frozen in liquid nitrogen. Then, the frozen samples were broken and fixed on a stub. Subsequently, the samples were coated with gold under a vacuum using a sputter coater (EM ACE600, Leica Microsystems, Wetzlar, Germany). The cross-sectional morphology of the samples was investigated using a JSM-7800F field emission scanning electron microscope (JEOL Ltd., Tokyo, Japan). The element composition and distribution of the composites were determined by energy dispersive spectroscopy coupled with SEM (SEM/EDS). Additionally, the morphology of samples in the swollen state was studied. The samples were soaked in DI water for 24 h. The swollen samples were freeze-dried overnight. After that, the freeze-dried samples were immersed in liquid nitrogen and then broken. They were sputter-coated with gold under vacuum, and their morphology was investigated through SEM. 

#### 2.5.4. Swelling Degree

The dried samples were weighed and immersed in DI water. The swollen samples were taken out and blotted onto filter paper to remove excess water on their surface. Then, the swollen samples were weighed. The swelling degree was determined by the following equation:

(4) $$Swelling\;degree\left(\%\right)=\frac{W_{s}-W_{d}}{W_{d}}\times 100$$

where W_d_ is the weight of the dried samples, and W_s_ is the weight of the swollen samples.

#### 2.5.5. Compressive Properties

The compressive properties of the samples were determined using a texture analyzer (TA.XT plus, Stable Micro Systems Ltd., Surrey, UK). Before testing, the samples were immersed in DI water for 24 h and cut into a cylindrical shape with a diameter of approximately 12 mm and a thickness of 5 mm. The swollen samples were placed on the plate and compressed to 80% strain. The measurement was run at a fixed strain rate of 0.05 mm/s. 

### 2.6. Dye Adsorption Ability

#### 2.6.1. Effect of Type of Adsorbent

The dye adsorption of different types of prepared samples was studied using methylene blue (MB) as a model dye. The samples were placed into 50 mL of a 50 mg/L dye solution. Dye solutions were taken out at various time intervals. The remaining dye concentration after adsorption was determined using UV-vis spectrophotometry (Cary Series, Agilent Technologies, Santa Clara, CA, USA) at a wavelength of 664 nm. The concentration of dye was obtained by comparison with the calibration curve of the MB solution (Figure S1, see Supplementary Materials). The dye adsorption ability was determined in terms of removal efficiency and adsorption capacity as follows [24]:

(5) $$Removal\;efficiency\left(\%\right)=\frac{C_{0}-C_{e}}{C_{0}}\times 100$$

(6) $$Q=\frac{C_{0}-C_{e}}{m}\times V$$

where C_0_ is the initial dye concentration in the solution (mg/L), C_e_ is the equilibrium dye concentration in the solution (mg/L), m is the weight of the dried samples (g), and V is the volume of the solution (L).

#### 2.6.2. Adsorption Kinetic

The experiment was carried out by adding the composites at different initial dye concentration levels (100–3000 mg/L). The amount of adsorbed dye was determined. To study dye adsorption kinetics, the pseudo-first-order and pseudo-second-order kinetic models are applied to fit the experimental data. The pseudo-first-order and pseudo-second-order kinetic models are presented in Equations (7) and (8), respectively, as follows [25]:

(7) $$\log\left(Q_{e}-Q_{t}\right)=logQ_{e}-\frac{k_{1}t}{2.303}$$

(8) $$\frac{t}{Q_{t}}=\frac{1}{k_{2}{Q_{e}}^{2}}+\frac{t}{Q_{e}}$$

where Q_e_ and Q_t_ represent the adsorption capacity of the dye at equilibrium (e) and time (t), respectively. The k_1_ and k_2_ are the rate constants of the pseudo-first-order and pseudo-second-order kinetic models, respectively. 

#### 2.6.3. Adsorption Isotherm

The adsorption isotherm was studied using Langmuir and Freundlich isotherm models [26]. The amount of adsorbed dye was examined by varying the initial concentrations of the dye solution in the range of 100–3000 mg/L. The Langmuir equation is presented by the following equation:

(9) $$\frac{C_{e}}{Q_{e}}=\frac{1}{Q_{m}K_{L}}+\frac{C_{e}}{Q_{m}}$$

where C_e_ is the concentration of dye remaining in the solution at equilibrium, Q_m_ is the maximum adsorption capacity, and K_L_ is the Langmuir constant. The essential parameter of Langmuir isotherm to classify adsorption behavior (R_L_) is shown by the following equation:

(10) $$R_{L}=\frac{1}{{1+K}_{L}C_{e}}$$

where the value of R_L_ is expressed as follows; R_L_ > 1 (Unfavorable), R_L_ = 1 (Linear), 0 < R_L_ < 1 (Favorable), and R_L_ = 0 (Irreversible).

For the Freundlich adsorption isotherm, it can be used to describe the heterogeneous surface with multilayer adsorption. The Freundlich equation is shown in Equation (11):

(11) $$logQ_{e}=logK_{F}+\frac{1}{n}logC_{e}$$

where K_F_ is the Freundlich constant, and n is the heterogeneity factor.

#### 2.6.4. Reusability

To study reusability, the adsorption was performed in 50 mL of a 100 mg/L dye solution. The used composites were regenerated by immersion in 0.5 M NaOH for 24 h and water for 24 h, respectively [12]. The adsorption test was conducted for 5 cycles using the same adsorbents. The removal efficiency was then calculated. Each sample was tested three times.

## 3. Results and Discussion

### 3.1. Characterization of (PAA-co-PAM)-Modified, Deproteinized, Natural Rubber

#### 3.1.1. Conversion, Grafting Efficiency and Grafting Percentage

The chemical compositions and properties of various types of (PAA-*co*-PAM)-modified DPNR are shown in Table 1. The conversion of all samples was found to range from 79.19 ± 4.40 to 83.34 ± 3.01%. When the monomer content was kept constant at 10 phr (sample N1-N4), the grafting efficiency and grafting percentage tended to increase with increasing MBA content. The MBA contents were varied from 0 to 1.00 by weight percentage of comonomer, resulting in an increase in grafting efficiency from 12.44 ± 1.36 to 48.48 ± 4.32% and a rise in grafting percentage from 1.24 ± 0.14 to 4.85 ± 0.43%. In this system, the grafting of PAA-*co*-PAM occurred at the natural rubber particle surface. The linkage of PAA-*co*-PAM chains during polymerization was obtained due to the addition of MBA, leading to the formation of a crosslinked network structure [27,28]. Consequently, the PAA-*co*-PAM chains can hold into the natural rubber-based structure, resulting in an increase in grafting efficiency and grafting percentage. The effect of the comonomer ratio of acrylic acid and acrylamide on the properties of the modified DPNR was also studied. The sample N4-N6 with different comonomer ratios exhibited good colloidal stability. At a ratio of 30:70 by weight percentage of acrylic acid and acrylamide, the grafting efficiency and grafting percentage were found to be 10.20 ± 2.33% and 1.02 ± 0.23%, respectively. When the amount of acrylic acid increased, the grafting efficiency also increased. The interaction between copolymers through hydrogen bonding of polyacrylic acid and polyacrylamide can lead to the formation of self-assembled structures, improving grafting efficiency [29,30,31]. However, some of the carboxylic acid groups of acrylic acid were neutralized with NaOH. Thus, the comonomer ratio of acrylic acid and acrylamide at 70:30 by weight percentage gave the highest grafting efficiency (48.48 ± 4.32%) and grafting percentage (4.85 ± 0.43%). Nevertheless, under the condition of 75:25 by weight percentage of acrylic acid and acrylamide, phase separation during polymerization was observed. This observation was probably due to the deterioration of the colloidal stability of rubber particles in an aqueous medium caused by high acidity [32]. The presence of PAA-*co*-PAM in the structure is a crucial factor that could affect the efficiency of materials for the dye adsorption process. The effect of monomer content on its properties was also studied by varying the monomer contents to 10 and 20 phr, denoted as N4 and N7, respectively. When the monomer content increased to 20 phr, the grafting efficiency and grafting percentage increased to 54.26 ± 1.55% and 10.63 ± 0.31%, respectively. The higher grafting efficiency indicated a greater amount of PAA-*co*-PAM formed in natural rubber-based materials, which is useful for applications.

#### 3.1.2. FTIR Analysis

**Figure 2:**
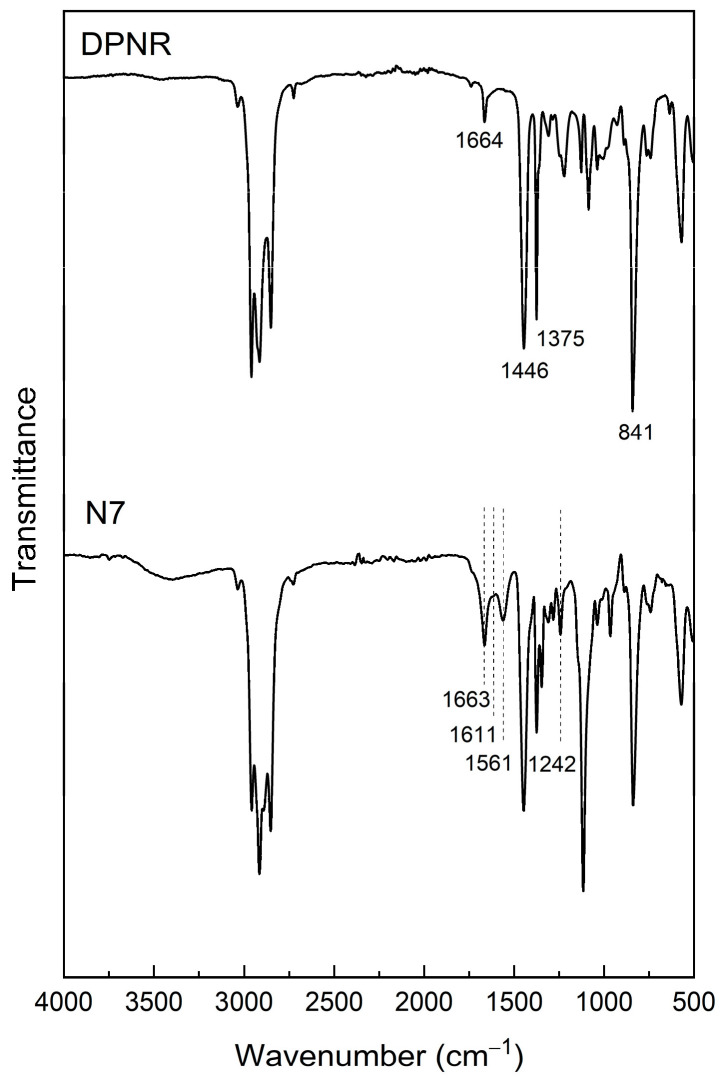
FTIR spectra of DPNR and (PAA-*co*-PAM)−modified DPNR.

The chemical functional groups in the sample structure were investigated by FTIR. The FTIR spectrum of the prepared (PAA-*co*-PAM)-modified DPNR (sample N7) after extraction with water was compared to that of the DPNR, as illustrated in Figure 2. For DPNR, peaks at 1664, 1446, 1375, and 841 cm^−^^1^ were observed, corresponding to C=C, -CH_2_, -CH_3_, and =CH, respectively [33]. These peaks are indicative of polyisoprene, which is the chemical constituent of natural rubber. After modification by graft copolymerization, sample N7 exhibited the characteristic peaks of DPNR and additional peaks of OH and NH stretching (3500–3200 cm^−^^1^), C=O stretching (1663 cm^−^^1^), NH bending (1611 cm^−^^1^), carboxylate (1561 cm^−^^1^), and C-O stretching (1242 cm^−^^1^) [34]. These additional signals were consistent with the functional groups of polyacrylic acid and polyacrylamide, confirming the presence of PAA-*co*-PAM after modification. These additional functional groups could play an important role in capturing adsorbate molecules [35]. 

### 3.2. Characterization of (PAA-co-PAM)-DPNR/Ag-TiO_2_ Composites

#### 3.2.1. Formation of Silver Nanoparticles

**Figure 3:**
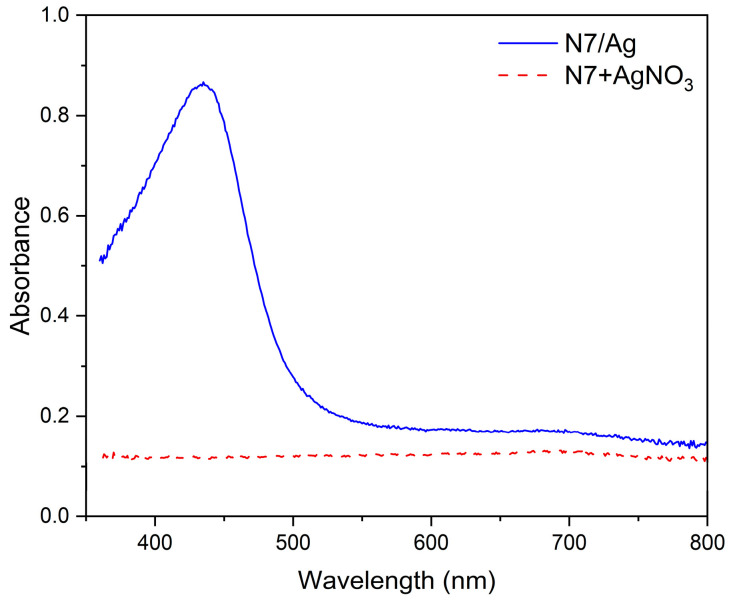
UV-vis spectra of N7/Ag composite compared to N7 mixed with AgNO_3_ solution.

**Figure 4:**
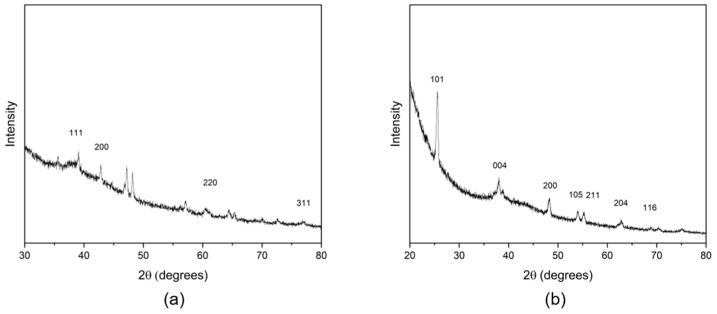
XRD patterns of (**a**) N7/Ag and (**b**) N7/Ag-Ti5 composites.

The (PAA-*co*-PAM)-modified DPNR was first prepared and then mixed with AgNO_3_ solution and titanium dioxide dispersion. The silver ions were coordinated with carboxylic acid groups containing (PAA-*co*-PAM)-DPNR to form the complex. The reduction of Ag^+^ ions to Ag^0^ was conducted under high temperatures and applied pressure. In this case, the natural rubber was modified by grafting with (PAA-*co*-PAM), resulting in the improved polarity of the natural rubber-based matrix. The addition of titanium dioxide dispersion could be compatible with the modified natural rubber. It can interact with the polymeric matrix through polar-polar interaction and hydrogen bonding between the hydroxylated titanium dioxide surface and carboxyl groups of PAA [36]. Therefore, the silver nanoparticles and titanium dioxide were dispersed in the natural rubber-based matrix. The titanium dioxide contents were varied as 1.0, 2.5, and 5.0 phr to produce the (PAA-*co*-PAM)-DPNR/Ag-TiO_2_ (N7/Ag-Ti) composites. Their properties were investigated and compared to samples prepared from modified natural rubber (N7), modified natural rubber containing silver nanoparticles (N7/Ag), and modified natural rubber filled with titanium dioxide (N7-Ti). The formulations and characteristics of all samples after drying are shown in Table 2. Sample N7 had a light-yellow color, while the color of the N7/Ag composite was an opaque dark brown, attributed to the occurrence of silver nanoparticles within the natural rubber-based composite [37]. In the case of the incorporation of titanium dioxide, the samples became lighter brown with an increasing amount of titanium dioxide. Meanwhile, N7-Ti without silver nanoparticles exhibited an opaque white color. The formation of silver nanoparticles was examined using a UV-vis spectrophotometer, as displayed in Figure 3. As seen from the results, the N7/Ag composite exhibits a strong absorption peak at around 435 nm, while this peak is not observed in the case of N7 mixed with AgNO_3_ solution. This peak corresponds to the surface plasmon resonance absorption of silver nanoparticles [38,39], indicating the formation of silver nanoparticles in natural rubber-based composites. Based on the estimation of the particle size of silver nanoparticles from UV-vis spectroscopy, as reported by Alim-Al-Razy et al. [40], it was found that the particle size of silver nanoparticles was approximately 65 nm. Furthermore, the XRD pattern of the N7/Ag composite shows diffraction peaks at 38.33°, 42.83°, 64.49°, and 77.21°, corresponding to the reflection plane indices of (111), (200), (220), and (311), respectively (Figure 4a). These correspond to the face-centered cubic (fcc) structure of metallic Ag. Similar observations have been reported in other research works [41,42]. For the N7/Ag-Ti5 composite (Figure 4b), the peaks at 25.60°, 38.01°, 48.21°, 54.02°, 55.36°, 62.93°, and 68.95° were observed, which were assigned to the (101), (004), (200), (105), (211), (204), and (116) of anatase TiO_2_, respectively [43]. However, the peaks corresponding to silver nanoparticles were less prominent. 

#### 3.2.2. Morphology

**Figure 5:**
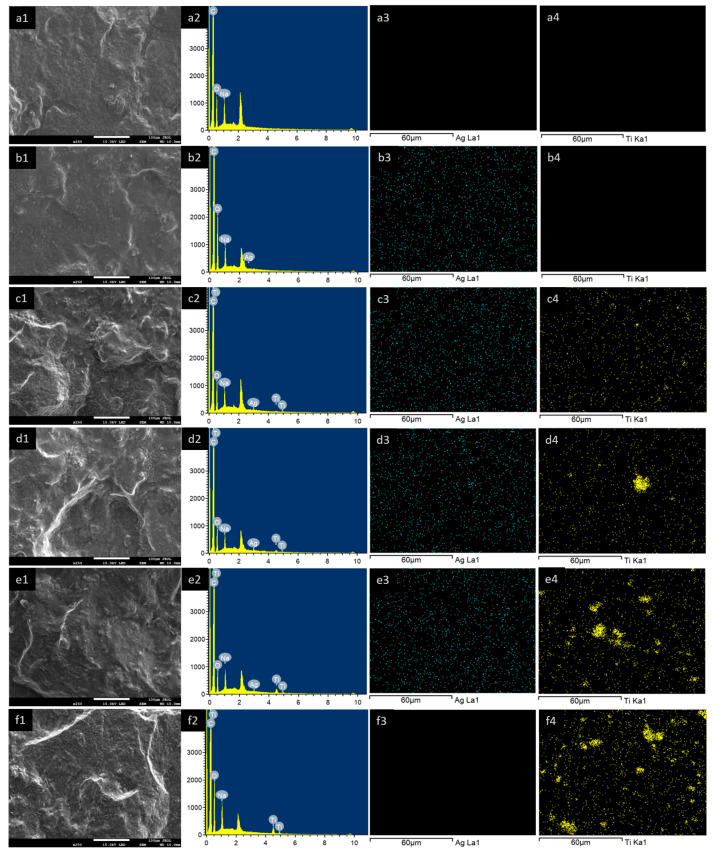
SEM images of cross-section surface in dry state (**first column**, at ×250 magnification), EDS spectra (**second column**), and EDS mappings (the green spots indicate silver and the yellow spots indicate titanium at **third** and **fourth columns**, respectively) of (**a1**–**a4**) N7; (**b1**–**b4**) N7/Ag; (**c1**–**c4**) N7/Ag-Ti1.0; (**d1**–**d4**) N7/Ag-Ti2.5; (**e1**–**e4**) N7/Ag-Ti5.0; (**f1**–**f4**) N7-Ti5.0.

**Figure 6:**
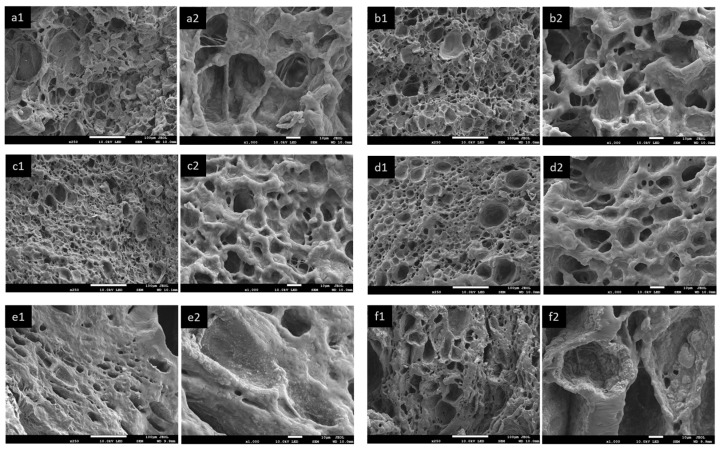
SEM images of cross-section surface of samples after immersion in water and freeze-drying with different magnifications (at ×250 and ×1000 magnifications) of (**a1**,**a2**) N7; (**b1**,**b2**) N7/Ag; (**c1**,**c2**) N7/Ag-Ti1.0; (**d1**,**d2**) N7/Ag-Ti2.5; (**e1**,**e2**) N7/Ag-Ti5.0; (**f1**,**f2**) N7-Ti5.0.

The cross-sectional morphology and EDS analysis of the (PAA-*co*-PAM)-DPNR/Ag-TiO_2_ composites in the solid stage are demonstrated in Figure 5. The dried samples were frozen in liquid nitrogen and then broken. All samples exhibited a dense solid cross-section surface, as observed from SEM images (first column). Upon adding titanium dioxide to the system, the roughness of the composites was enhanced compared to N7 and N7/Ag. Their EDS spectra are also displayed in the second column. The characteristic signals of C, O, and Na appeared for the sample N7. An additional peak corresponding to Ag was found in the case of N7/Ag and (PAA-*co*-PAM)-DPNR/Ag-TiO_2_ composites. For (PAA-*co*-PAM)-DPNR/Ag-TiO_2_ composites, additional signals corresponding to Ti were also found. The elemental compositions of the composites from the EDS analysis are reported in Table 3. It was clearly observed that the weight and atomic percentages of titanium increased relative to the amount of titanium dioxide. The EDS mappings corresponding to Ag and Ti are shown in the third and fourth columns, respectively. The good distribution of Ag in the matrix is observed, and the presence of Ti dispersed in the natural rubber-based matrix was also noted. The size of the titanium dioxide particles was about 208.56 ± 39.77 nm. However, with an increase in the titanium dioxide content, there was an observed increase in the extent of particle agglomeration, with sizes in the range of 0.82–6.43 µm. In addition, the cross-sectional morphology of samples after immersion in water was also examined. The samples were soaked in water and subjected to freeze-drying. Their cross-section surfaces were also observed from SEM images, as shown in Figure 6. It was noted that the morphology of all samples changed from a dense solid surface to a porous structure after soaking in water. The formation of the porous structure could be explained by the penetration of water molecules into the crosslinked sample structure. After freeze-drying, the trapped water sublimated, resulting in the porous morphology [44,45]. Samples with many pores in their structure had advantages for applications. In the adsorption process, adsorbate molecules were initially absorbed on the outer surface and then diffused into the inner layer [46]. Therefore, the formation of a porous framework could provide a high surface area for binding with other substances in an aqueous solution, offering benefits for the adsorption process. Moreover, roughness at the pore wall was observed when titanium dioxide was introduced. In particular, when the amount of titanium dioxide increased to 5.0 phr, titanium dioxide could appear on the wall surface. 

#### 3.2.3. Swelling Degree

**Figure 7:**
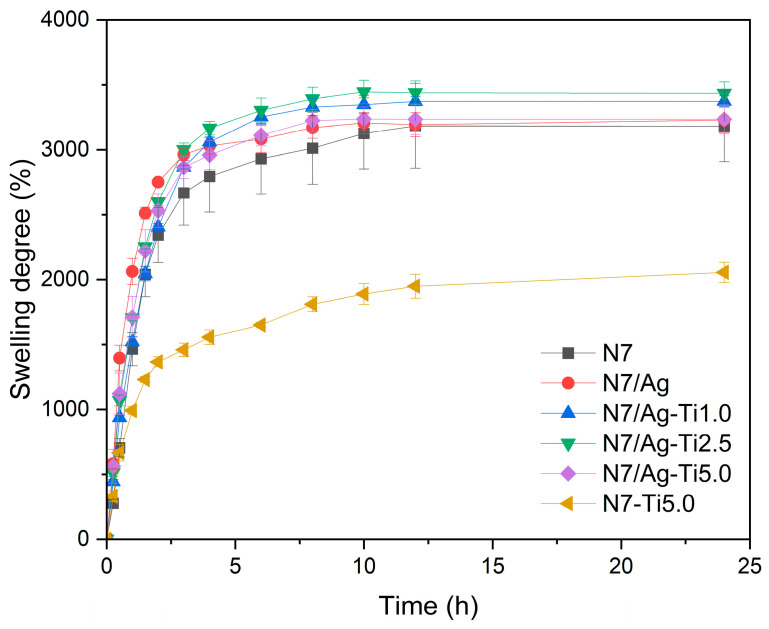
Swelling degree of the (PAA-*co*-PAM)-DPNR/Ag-TiO_2_ composites after immersion in DI water for 24 h.

Figure 7 shows the swelling degree of the composites against immersion time. As a result, the swelling degree increased with the immersion time of the samples until reaching the equilibrium point. The swelling degree of N7 was found to be 3180.6 ± 273.0% after 24 h. In this case, the main matrix is natural rubber modified with hydrophilic PAA-*co*-PAM, allowing water to diffuse into its structure. For the composites comprising silver nanoparticles and titanium dioxide, there was a slight increase in swelling degree (3374.5 ± 40.6 to 3434.2 ± 88.1%) when the titanium dioxide was 1.0–2.5 phr. This increase is attributed to the enhancement of the polarity of the composites. When the titanium dioxide content changed to 5.0 phr, the swelling degree decreased. This decrease may be due to the agglomeration of particles, as seen from the SEM images. It was observed that the composite filled with titanium dioxide without silver nanoparticles at the same amount of titanium dioxide (5.0 phr) exhibited a decrease in swelling degree (2055.8 ± 76.8%) compared to N7. This is probably because of the weak interaction with the main matrix, and a large number of titanium dioxide particles were deposited on the wall of the matrix, blocking water absorption. The swelling degree of the composite containing only silver nanoparticles remained high swelling degree (3229.0 ± 98.3%). It can be explained by Hou et al., where a stable PAA/silver nanoparticle gel was obtained due to the coordination between PAA and silver ion as well as a self-assembly process [47]. Therefore, the strong interaction between PAA and silver plays an important role in preparing materials with good structural stability and high water absorption. 

#### 3.2.4. Compressive Properties

**Figure 8:**
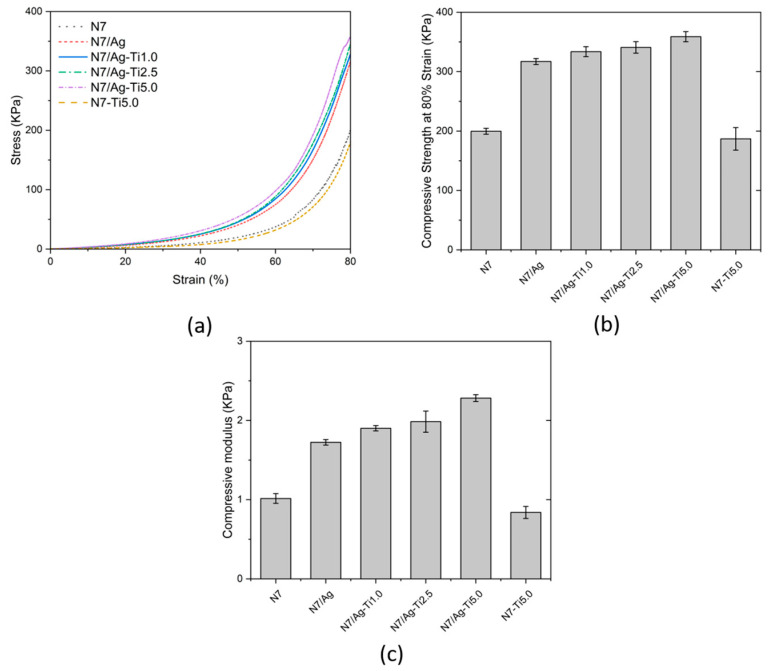
(**a**) Stress-strain curve measured in the swollen state, (**b**) compressive strength at 80% strain, (**c**) compressive modulus at 0–10% strain of the (PAA-*co*-PAM)-DPNR/Ag-TiO_2_ composites.

The mechanical properties of the prepared composites were investigated through compression testing. As these composites were employed as adsorbents in an aqueous medium, their compressive properties were examined under swollen conditions. The stress-strain curve of the swollen samples is displayed in Figure 8a. It was observed that the stress increased when the compression strain increased. The result revealed that the samples had high endurance to external compressive loading. The compressive strength at 80% strain and compressive modulus are shown in Figure 8b,c, respectively. The compressive strength and compressive modulus of N7 were found to be 199.65 ± 5.01 and 1.015 ± 0.062 KPa, respectively. The samples incorporating silver nanoparticles exhibited high compressive strength and modulus. Thus, the coordination of carboxylic groups in the composites and silver ions contributes to the enhancement of mechanical properties through the formation of a strong network inside the structure [48]. For the N7/Ag composite, its compressive strength and compressive modulus increased to 317.04 ± 5.05 and 1.724 ± 0.035 KPa, respectively. When titanium dioxide was introduced, their compressive strength values slightly increased. They seemed to increase with the increase in titanium dioxide contents. The compressive strength increased from 323.18 ± 10.41 to 347.91 ± 11.42 KPa, and the compressive modulus increased from 1.902 ± 0.033 to 2.283 ± 0.043 KPa when the titanium dioxide increased from 1.0 to 5.0 phr. It was observed that the compressive strength and compressive modulus of the N7/Ag-Ti5.0 composite increased by 1.80 and 2.25 times compared to N7. However, the compressive strength of N7-Ti5 slightly decreased from N7. This may be due to the weak interaction between modified natural rubber and titanium dioxide. Therefore, the combination of silver nanoparticles and titanium dioxide in the composites could improve the mechanical properties of natural rubber-based materials. Moreover, the samples could maintain their integrity after applying external force without fracturing. Considering the sample structure, the main component of these samples is composed of natural rubber, which exhibits high elasticity [49]. Natural rubber-based materials can quickly return to their original or similar sizes after deformation. This indicates that the prepared composites exhibited good structural stability and mechanical properties, which would be effective for various applications. 

### 3.3. Dye Adsorption Studies

#### 3.3.1. Effect of Type of (PAA-*co*-PAM)-DPNR

**Figure 9:**
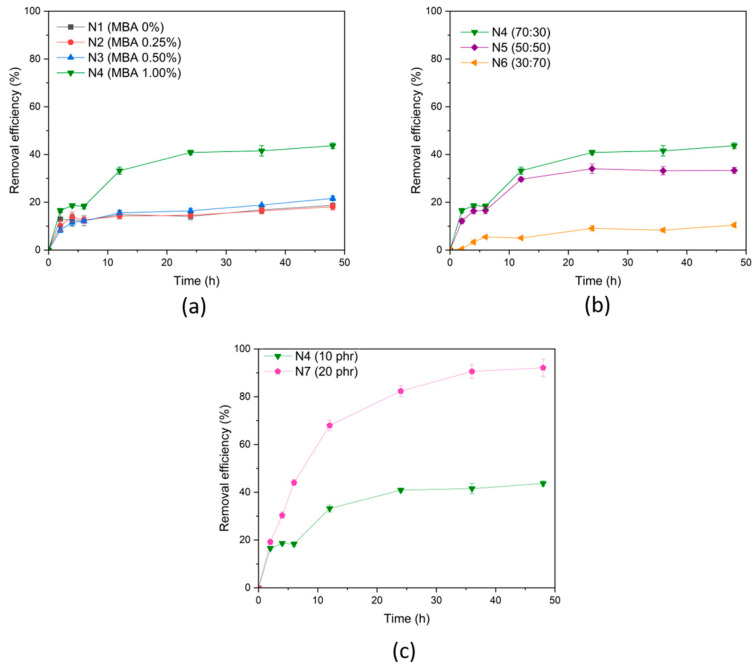
Effect of (**a**) MBA crosslinker content, (**b**) weight ratio of acrylic acid and acrylamide, and (**c**) monomer content on dye adsorption ability.

The dye adsorption ability of different types of (PAA-*co*-PAM)-DPNR was examined, as shown in Figure 9. The study of dye adsorption using methylene blue (MB) as a dye model was conducted. Based on the characteristics of the samples, they exhibited a porous structure after immersion in an aqueous medium so that the dye solution could diffuse into the sample structure. According to the chemical structure of (PAA-*co*-PAM)-DPNR, it possesses specific functional groups with the ability to adsorb MB molecules [50,51]. Thus, these materials could effectively remove MB molecules from the aqueous medium. The effect of MBA content on the dye adsorption ability was studied, as displayed in Figure 9a. In the 0–24 h range, the removal efficiency values increased with immersion time due to the abundance of active sites that could interact with dye molecules in the initial period. After 24 h, the removal efficiency values remained constant. It was observed that the (PAA-*co*-PAM)-modified DPNR with a higher content of MBA crosslinking agent exhibited a higher removal efficiency. The removal efficiency values increased from 14.13 ± 1.51 to 40.87 ± 0.92%, while the MBA content increased from 0 to 1.00%. This can be explained by the addition of MBA, which improves the ability to hold (PAA-*co*-PAM) on its structure, increasing dye adsorption ability. In addition, the (PAA-*co*-PAM)-modified DPNR with the weight ratio of acrylic acid and acrylamide at 70:30 showed higher removal efficiency than those of other conditions, as shown in Figure 9b. Under these conditions, the obtained (PAA-co-PAM) modified DPNR had high grafting efficiency, indicating a significant number of active functional groups available to adsorb dye molecules. Moreover, it was probably because the interaction of carboxylate groups and dye was stronger than that of -NH_2_ groups [52]. Furthermore, the result from Figure 9c shows that the (PAA-*co*-PAM)-modified DPNR prepared using 20 phr of monomer exhibited higher removal efficiency than that of 10 phr of monomer. Its removal efficiency was found to be 88.49 ± 3.21% after being immersed for 24 h. These indicate that the efficiency of dye adsorption depends on the amount of PAA-*co*-PAM present in the structure.

#### 3.3.2. Effect of Type of (PAA-*co*-PAM)-DPNR/Ag-TiO_2_ Composites

**Figure 10:**
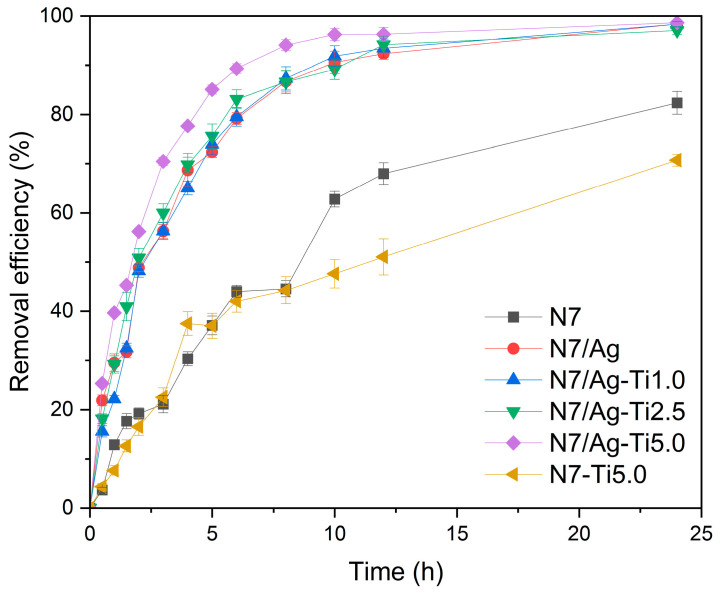
Effect of type of adsorbents on the removal efficiency after being immersed in the dye solution.

The comparison in dye adsorption ability of the prepared composites is illustrated in Figure 10. In the initial stage, the removal efficiency increased with the immersion time due to numerous available active sites interacting with dye molecules. After that, the removal efficiency gradually reached a constant value. At 12 h, the N7 had a removal efficiency of 67.96 ± 2.20%. The removal efficiency was enhanced when using the (PAA-*co*-PAM)-modified natural rubber consisting of silver nanoparticles and titanium dioxide. The removal efficiency of N7/Ag composite was 92.37 ± 1.18% after being immersed in dye solution for 12 h. As seen from the results, the N7/Ag-TiO_2_ composites exhibited higher removal efficiency than N7 and N7/Ag and seemed to increase with the increase in titanium dioxide content. The removal efficiency values of N7/Ag-TiO_2_ composites were found to be 93.47 ± 1.87, 94.19 ± 1.71, and 96.28 ± 1.38% when the titanium dioxide content was adjusted to 1.0, 2.5, and 5.0 phr, respectively. However, the modified natural rubber containing only titanium dioxide did not show an increase in adsorption efficiency. Generally, titanium dioxide is used as a photocatalyst for the photocatalytic degradation of various pollutants, capable of degrading chemical contaminants. However, its performance is notably enhanced under UV radiation [53]. The synergistic removal efficiency for modified natural rubber containing silver nanoparticles/titanium dioxide was observed. This is probably because the incorporation of silver nanoparticles can enhance the response to visible light [54]. The transfer of electrons from Ag to TiO_2_ is mediated by localized surface plasmon resonance, which then reacts with adsorbed molecules [55]. Additionally, the holes in plasmonic silver nanoparticles can capture conduction electrons of TiO_2_, therefore preventing the charge recombination phenomenon [56]. Therefore, the presence of silver nanoparticles and titanium dioxide distributed in the natural rubber-based matrix promotes dye-removal efficiency. 

#### 3.3.3. Adsorption Kinetic

**Figure 11:**
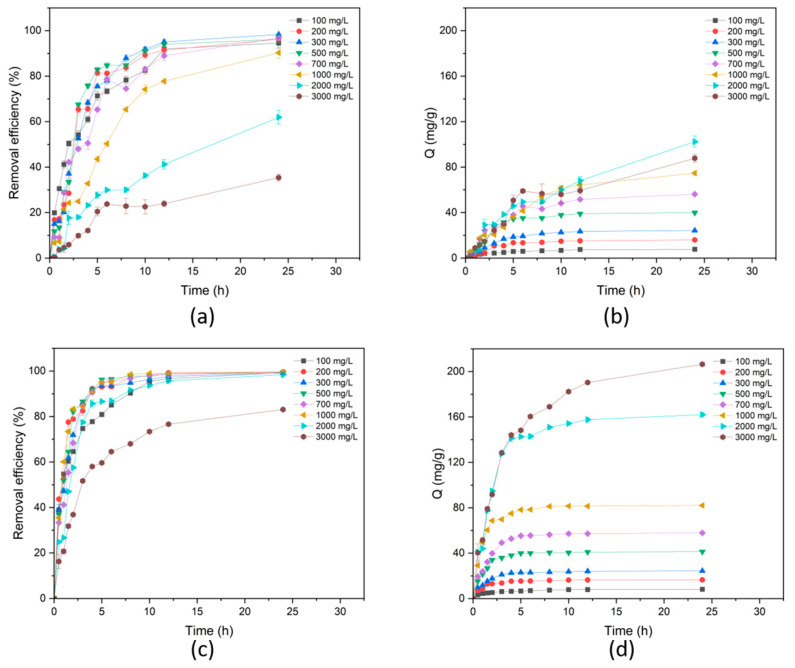
Dye adsorption with different initial dye concentrations of N7; (**a**) removal efficiency and (**b**) adsorption capacity, and N7/Ag-Ti5.0; (**c**) removal efficiency and (**d**) adsorption capacity.

The dye adsorption kinetic was studied using N7 and N7/Ag-Ti5.0 composites. Figure 11 shows the removal efficiency and adsorption capacity of both samples at different MB concentrations. It can be observed that the equilibrium-adsorption capacity (Q_e_) of N7 and N7/Ag-Ti5.0 increased with increasing dye concentration. The N7/Ag-Ti5.0 composite exhibited a higher rate of dye adsorption compared to N7. The Q_e_ value of N7/Ag-Ti5.0 was higher than that of N7 at the same initial concentration of dye. To study the dye adsorption kinetic, the pseudo-first-order and pseudo-second-order models were applied to fit the experimental data. Table 4 displays the calculated kinetic parameters of MB onto N7 and N7/Ag-Ti5.0 at different initial dye concentrations. As seen from the results, the pseudo-second-order kinetic model provided better agreement due to higher correlation coefficient (R^2^) values. The adsorption capacity values of the experiment were also close to those of theoretical adsorption capacity calculated from the pseudo-second-order kinetic model. These indicate that the adsorption process occurred through chemisorption [57]. 

#### 3.3.4. Adsorption Isotherm

The plots of the equilibrium-adsorption capacity of MB on N7 and N7/Ag-Ti5.0 are shown in Figure S2 (see Supplementary Materials). The adsorption of MB using N7 gave a maximum adsorption capacity of 102.35 mg/g, while the maximum adsorption capacity value increased to 206.42 mg/g for N7/Ag-Ti5.0. It can be explained by the incorporation of silver nanoparticles/titanium dioxide, which enhances the physical properties of the composites, such as water absorption, mechanical properties, and structural stability. Moreover, the removal of dye occurred through the binding with functional groups present in the natural rubber-based matrix and the catalytic activity due to the existence of silver nanoparticles/titanium dioxide. Based on its characteristics and adsorption capacity, the N7/Ag-Ti5.0 composite exhibited good performance for the dye-removal process. Additionally, the Langmuir and Freundlich isotherm models were applied to fit the adsorption data to study the adsorption phenomenon. Their adsorption isotherm parameters of MB are listed in Table 5. The results show that the correlation coefficient (R^2^) values of the Langmuir isotherm model were higher than those of the Freundlich isotherm model. The adsorption isotherm is best fitted with the Langmuir isotherm model. It indicated that the dye molecules were adsorbed on the composites, followed by monolayer binding [58]. The interaction between active sites of adsorbents and MB molecules occurred through hydrogen bonding from -NH_2_ groups of PAM and N atoms of MB molecules [12]. In addition, the carboxylate ions of PAA can also interact with positively charged MB through electrostatic attraction [59]. According to the Langmuir equation, the essential factor of the Langmuir isotherm (R_L_) corresponds to the type of adsorption behavior. They were in the range of 0–1 for both N7 and N7/Ag-Ti5.0, suggesting a favorable adsorption process. 

#### 3.3.5. Reusability

**Figure 12:**
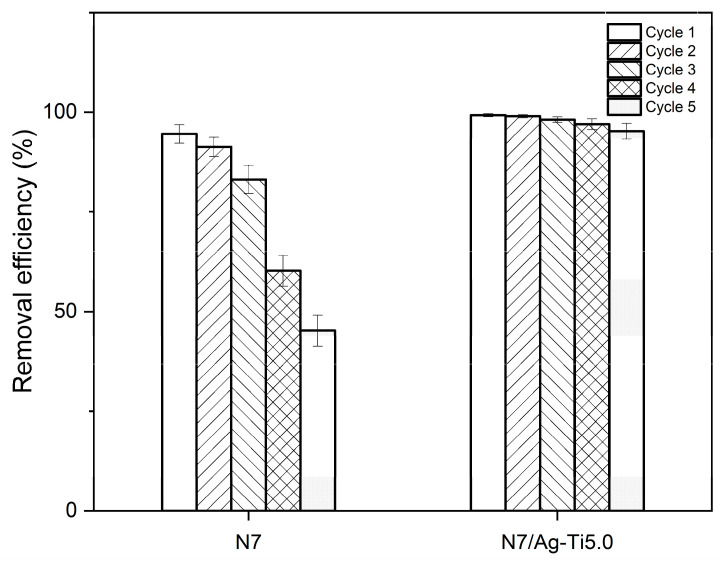
Removal efficiencies of the samples after immersion in dye solution with N7 and N7/Ag-Ti5.0 for five cycles.

The reusability of the prepared composites is also an important factor for evaluating the performance of the products in applications. Figure 12 shows the adsorption cycles of N7/Ag-Ti5.0 compared to N7 for 5 cycles. In the first adsorption cycle, the removal efficiency values of N7 and N7/Ag-Ti5.0 were found to be 94.53 ± 2.32 and 99.27 ± 0.38%, respectively. It can be clearly seen that the removal efficiency decreased when the number of regeneration cycles increased for N7. Its removal efficiency decreased by 52.15%, reaching a value of 45.23 ± 3.92% at cycle 5. However, the N7/Ag-Ti5.0 composite was able to maintain its removal efficiency. Its removal efficiency was around 91.52 ± 1.98% after repeated use for 5 cycles. Therefore, the prepared composites had potential as adsorbents for the highly efficient removal of dye from aqueous solution and reusability. 

## 4. Conclusions

The poly(acrylic acid-*co*-acrylamide) modified, deproteinized, and natural rubber composites consisting of silver nanoparticles and titanium dioxide were successfully prepared and utilized for the dye-removal process in this study. The (PAA-*co*-PAM)-DPNR was prepared by emulsion-graft copolymerization and subsequently mixed with AgNO_3_ solution and titanium dioxide dispersion. The formation of silver nanoparticles occurred under high temperatures and applied pressure without the use of a strong reducing agent, as confirmed by UV-vis spectrophotometry and XRD. The composites allowed water diffusion into their structure, resulting in a porous morphology that provided a high specific area for capturing dye molecules. The presence of silver nanoparticles and titanium dioxide improved the water absorption ability and mechanical properties of the composites. The compressive strength and compressive modulus of (PAA-*co*-PAM)-DPNR/Ag-TiO_2_ composites with 5.0 phr of titanium dioxide increased by 1.80- and 2.25-fold compared to (PAA-*co*-PAM)-DPNR. The composites could maintain their integrity without fracturing after immersion in water and application of external force. The (PAA-*co*-PAM)-DPNR possessed functional groups to adsorb dye molecules, with a maximum adsorption capacity of 102.35 mg/g. The incorporation of silver nanoparticles and titanium dioxide increased the dye-removal ability, with the maximum adsorption capacity of (PAA-*co*-PAM)-DPNR/Ag-TiO_2_ composites reaching 206.42 mg/g. The kinetic of dye removal was fitted with the pseudo-second-order kinetic model, and the adsorption behavior was found to agree with the Langmuir isotherm model. The composites could be reused with a removal efficiency of more than 90%, even after repeated use for 5 cycles. Therefore, these composites demonstrated high removal performance, which would be useful for the dye-removal process in aqueous conditions. 

## Figures and Tables

**Table 1:** Conversion, grafting efficiency, and grafting percentage of (PAA-*co*-PAM)-modified DPNR.

Samples	Monomer Contents (phr) *	AA:AM	MBA Contents ** (%)	Conversion (%)	Grafting Efficiency (%)	Grafting Percentage (%)
N1	10	70:30	0	81.51 ± 2.04	12.44 ± 1.36	1.24 ± 0.14
N2	10	70:30	0.25	80.69 ± 0.14	15.60 ± 2.65	1.56 ± 0.27
N3	10	70:30	0.50	79.19 ± 4.40	27.60 ± 0.69	2.76 ± 0.07
N4	10	70:30	1.00	82.66 ± 2.38	48.48 ± 4.32	4.85 ± 0.43
N5	10	50:50	1.00	81.91 ± 3.23	19.30 ± 3.32	1.93 ± 0.33
N6	10	30:70	1.00	80.62 ± 2.49	10.20 ± 2.33	1.02 ± 0.23
N7	20	70:30	1.00	83.34 ± 3.01	54.26 ± 1.55	10.63 ± 0.31

* part per hundred rubber; ** percentage by weight of monomer added.

**Table 2:** Composition of ingredients and physical appearances of the (PAA-*co*-PAM)-DPNR/Ag-TiO_2_ composites.

Samples	N7	N7/Ag	N7/Ag-Ti1.0	N7/Ag-Ti2.5	N7/Ag-Ti5.0	N7-Ti5.0
(PAA-*co*-PAM)-DPNR (phr)	100	100	100	100	100	100
AgNO_3_ (phr)	0	0.5	0.5	0.5	0.5	0
TiO_2_ (phr)	0	0	1.0	2.5	5.0	5.0
Characteristics	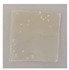	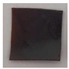	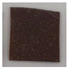	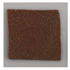	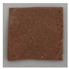	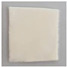

**Table 3:** Elemental composition from EDS results of the (PAA-*co*-PAM)-DPNR/Ag-TiO_2_ composites.

Samples	Weight (%)					Atomic (%)				
	C	O	Na	Ag	Ti	C	O	Na	Ag	Ti
N7	83.13	14.79	2.08	0.00	0.00	87.21	11.65	1.14	0.00	0.00
N7/Ag	75.98	21.95	1.74	0.32	0.00	81.34	17.64	0.97	0.04	0.00
N7/Ag-Ti1.0	83.73	14.18	1.53	0.43	0.13	87.90	11.17	0.84	0.05	0.04
N7/Ag-Ti2.5	84.81	12.72	1.67	0.47	0.33	88.93	10.01	0.91	0.06	0.09
N7/Ag-Ti5.0	87.66	9.31	1.70	0.55	0.79	91.51	7.30	0.93	0.06	0.21
N7-Ti5.0	78.90	18.24	1.93	0.00	0.94	84.08	14.60	1.07	0.00	0.25

**Table 4:** Pseudo-first-order kinetic and pseudo-second-order kinetic parameters of dye onto N7 and N7/Ag-Ti5.0.

Initial Dye Concentration (mg/L)	Q_e_(exp) (mg/g)	Pseudo-First-Order Kinetic Model			Pseudo-Second-Order Kinetic Model		
		k_1_ (L/h)	Q_e_(theo) (mg/g)	R^2^	k_2_ (g/mg·h)	Q_e_(theo) (mg/g)	R^2^
N7							
100	7.78	0.0991	8.19	0.9250	0.0482	8.60	0.9974
200	15.97	0.0816	15.90	0.6289	0.0133	19.51	0.9519
300	24.28	0.1194	24.07	0.7490	0.0076	30.30	0.9495
500	39.95	0.1190	36.00	0.8154	0.0053	49.26	0.9357
700	56.24	0.0840	54.98	0.4931	0.0022	74.07	0.8769
1000	74.68	0.0675	77.87	0.9721	0.0007	117.65	0.8394
2000	102.35	0.0193	197.60	0.6736	0.0006	140.85	0.9060
3000	87.77	0.0685	74.54	0.8757	0.0005	140.85	0.8311
N7/Ag-Ti5.0							
100	8.20	0.2676	7.24	0.9765	0.1034	8.59	0.9991
200	16.54	0.1500	16.00	0.9324	0.1013	17.06	0.9994
300	24.43	0.1579	22.74	0.9021	0.0519	25.38	0.9990
500	41.25	0.2702	40.33	0.9704	0.0375	42.74	0.9989
700	57.89	0.0779	57.64	0.9511	0.0181	60.98	0.9973
1000	82.04	0.0791	72.00	0.9308	0.0208	84.75	0.9993
2000	162.02	0.1152	162.34	0.9680	0.0036	175.44	0.9933
3000	206.42	0.0980	198.00	0.9855	0.0015	232.56	0.9976

**Table 5:** Langmuir and Freundlich isotherm parameters of the adsorption of dye onto N7 and N7/Ag-Ti5.0.

Samples	Langmuir Adsorption Isotherm				Freundlich Adsorption Isotherm		
	Q_m_ (mg/g)	K_L_ (L/mg)	R_L_	R^2^	K_F_ (mg/g)(L/mg)^1/n^	n	R^2^
N7							
	90.09	0.0982	0.0053	0.9963	0.0131	0.4604	0.6933
N7/Ag-Ti5.0							
	208.33	0.1387	0.0142	0.9998	0.0010	0.5673	0.7406

## Data Availability

Data are contained within the article.

## Supplementary Materials

The following supporting information can be downloaded at: https://www.mdpi.com/article/10.3390/polym16010092/s1, Figure S1: Calibration curve of MB solution.; Figure S2: Equilibrium-adsorption capacity as a function of equilibrium dye concentration of (a) N7, and (b) N7/Ag-Ti5.0.

## References

[1] Cook A.B., Perrier S. (2020). Branched and Dendritic Polymer Architectures: Functional Nanomaterials for Therapeutic Delivery. Adv. Funct. Mater..

[2] Kavand A., Anton N., Vandamme T., Serra C.A., Chan-Seng D. (2020). Synthesis and functionalization of hyperbranched polymers for targeted drug delivery. J. Control. Release.

[3] Xiao F., Cheng J., Cao W., Yang C., Chen J., Luo Z. (2019). Removal of heavy metals from aqueous solution using chitosan-combined magnetic biochars. J. Colloid Interface Sci..

[4] Zhang X., Zheng J., Jin P., Xu D., Yuan S., Zhao R., Depuydt S., Gao Y., Xu Z.-L., Van der Bruggen B. (2022). A PEI/TMC membrane modified with an ionic liquid with enhanced permeability and antibacterial properties for the removal of heavy metal ions. J. Hazard. Mater..

[5] Yuan Z., Wang J., Wang Y., Liu Q., Zhong Y., Wang Y., Li L., Lincoln S.F., Guo X. (2019). Preparation of a poly (acrylic acid) based hydrogel with fast adsorption rate and high adsorption capacity for the removal of cationic dyes. RSC Adv..

[6] Huang T., Shao Y.-W., Zhang Q., Deng Y.-F., Liang Z.-X., Guo F.-Z., Li P.-C., Wang Y. (2019). Chitosan-Cross-Linked Graphene Oxide/Carboxymethyl Cellulose Aerogel Globules with High Structure Stability in Liquid and Extremely High Adsorption Ability. ACS Sustain. Chem. Eng..

[7] Singha N.R., Roy C., Mahapatra M., Dutta A., Deb Roy J.S., Mitra M., Chattopadhyay P.K. (2019). Scalable Synthesis of Collagenic-Waste and Natural Rubber-Based Biocomposite for Removal of Hg(II) and Dyes: Approach for Cost-Friendly Waste Management. ACS Omega.

[8] Guerra N.B., Sant’Ana Pegorin G., Boratto M.H., de Barros N.R., de Oliveira Graeff C.F., Herculano R.D. (2021). Biomedical applications of natural rubber latex from the rubber tree *Hevea brasiliensis*. Mater. Sci. Eng. C.

[9] Mosaku A.M., Akinlabi A.K., Sojinu O.S., Arifalo M.K.O., Falomo A.A., Oladipo G., Oni S., Falope F.Y., Ilesanmi N.Y., Diayi V.N. (2021). Adsorptive Remediation of Oil Spill Contaminated Water Using Chitosan Modified Natural Rubber as Adsorbent. Chem. Afr..

[10] Jermjun K., Khumho R., Thongoiam M., Yousatit S., Yokoi T., Ngamcharussrivichai C., Nuntang S. (2023). Natural Rubber/Hexagonal Mesoporous Silica Nanocomposites as Efficient Adsorbents for the Selective Adsorption of (-)-Epigallocatechin Gallate and Caffeine from Green Tea. Molecules.

[11] Inphonlek S., Jarukumjorn K., Chumsamrong P., Ruksakulpiwat C., Ruksakulpiwat Y. (2023). Preparation of Crosslinked Poly(acrylic acid-co-acrylamide)-Grafted Deproteinized Natural Rubber/Silica Composites as Coating Materials for Controlled Release of Fertilizer. Polymers.

[12] Maijan P., Junlapong K., Arayaphan J., Khaokong C., Chantarak S. (2021). Synthesis and characterization of highly elastic superabsorbent natural rubber/polyacrylamide hydrogel. Polym. Degrad. Stab..

[13] Mohamad Idris N.H., Rajakumar J., Cheong K.Y., Kennedy B.J., Ohno T., Yamakata A., Lee H.L. (2021). Titanium Dioxide/Polyvinyl Alcohol/Cork Nanocomposite: A Floating Photocatalyst for the Degradation of Methylene Blue under Irradiation of a Visible Light Source. ACS Omega.

[14] Al-Madanat O., AlSalka Y., Ramadan W., Bahnemann D.W. (2021). TiO_2_ Photocatalysis for the Transformation of Aromatic Water Pollutants into Fuels. Catalysts.

[15] Suriyachai N., Chuangchote S., Laosiripojana N., Champreda V., Sagawa T. (2020). Synergistic Effects of Co-Doping on Photocatalytic Activity of Titanium Dioxide on Glucose Conversion to Value-Added Chemicals. ACS Omega.

[16] Wongpreecha J., Polpanich D., Suteewong T., Kaewsaneha C., Tangboriboonrat P. (2018). One-pot, large-scale green synthesis of silver nanoparticles-chitosan with enhanced antibacterial activity and low cytotoxicity. Carbohydr. Polym..

[17] Ni Z., Wang Z., Sun L., Li B., Zhao Y. (2014). Synthesis of poly acrylic acid modified silver nanoparticles and their antimicrobial activities. Mater. Sci. Eng. C.

[18] Ghorbanloo M., Nosrati Fallah H. (2020). Silver nanoparticle embedded anionic crosslinked copolymer hydrogels: An efficient catalyst. J. Porous Mater..

[19] Fahmy A., Agudo Jácome L., Schönhals A. (2020). Effect of Silver Nanoparticles on the Dielectric Properties and the Homogeneity of Plasma Poly(acrylic acid) Thin Films. J. Phys. Chem. C.

[20] Kawahara S., Klinklai W., Kuroda H., Isono Y. (2004). Removal of proteins from natural rubber with urea. Polym. Adv. Technol..

[21] Pipertzis A., Kafetzi M., Giaouzi D., Pispas S., Floudas G.A. (2022). Grafted Copolymer Electrolytes Based on the Poly(acrylic acid-co-oligo ethylene glycol acrylate) (P(AA-co-OEGA)) Ion-Conducting and Mechanically Robust Block. ACS Appl. Polym. Mater..

[22] Rimdusit N., Jubsilp C., Mora P., Hemvichian K., Thuy T.T., Karagiannidis P., Rimdusit S. (2021). Radiation Graft-Copolymerization of Ultrafine Fully Vulcanized Powdered Natural Rubber: Effects of Styrene and Acrylonitrile Contents on Thermal Stability. Polymers.

[23] Shi Z., Jia C., Wang D., Deng J., Xu G., Wu C., Dong M., Guo Z. (2019). Synthesis and characterization of porous tree gum grafted copolymer derived from *Prunus cerasifera* gum polysaccharide. Int. J. Biol. Macromol..

[24] Abdel-Raouf M.E.-S., Farag R.K., Farag A.A., Keshawy M., Abdel-Aziz A., Hasan A. (2023). Chitosan-Based Architectures as an Effective Approach for the Removal of Some Toxic Species from Aqueous Media. ACS Omega.

[25] Mokhtari A., Sabzi M., Azimi H. (2021). 3D porous bioadsorbents based on chitosan/alginate/cellulose nanofibers as efficient and recyclable adsorbents of anionic dye. Carbohydr. Polym..

[26] Singh N., Riyajuddin S., Ghosh K., Mehta S.K., Dan A. (2019). Chitosan-Graphene Oxide Hydrogels with Embedded Magnetic Iron Oxide Nanoparticles for Dye Removal. ACS Appl. Nano Mater..

[27] Cao W., Ding P., Ding Q., Liu C., Yu W., Hu L. (2023). Shear Responsive Gelation of Aqueous Polyacrylic Acid-co-polyacrylamide: Molecular Mechanism and Tribological Applications. ACS Appl. Polym. Mater..

[28] Arunbabu D., Shahsavan H., Zhang W., Zhao B. (2013). Poly(AAc-co-MBA) Hydrogel Films: Adhesive and Mechanical Properties in Aqueous Medium. J. Phys. Chem. B.

[29] Sennakesavan G., Mostakhdemin M., Dkhar L.K., Seyfoddin A., Fatihhi S.J. (2020). Acrylic acid/acrylamide based hydrogels and its properties—A review. Polym. Degrad. Stab..

[30] Konko I., Guriyanova S., Boyko V., Sun L., Liu D., Reck B., Men Y. (2019). Role of the Hydrophilic Latex Particle Surface in Water Diffusion into Films from Waterborne Polymer Colloids. Langmuir.

[31] Jing Z., Xu A., Liang Y.-Q., Zhang Z., Yu C., Hong P., Li Y. (2019). Biodegradable Poly(acrylic acid-co-acrylamide)/Poly(vinyl alcohol) Double Network Hydrogels with Tunable Mechanics and High Self-healing Performance. Polymers.

[32] Ishiaku U.S., Shaharum A., Ismail H., Ishak Z.A.M. (1998). The effect of an epoxidized plasticizer on the thermo-oxidative ageing of poly(vinyl chloride)/epoxidized natural rubber thermoplastic elastomers. Polymer Int..

[33] Chalid M., Husnil Y.A., Puspitasari S., Cifriadi A. (2020). Experimental and Modelling Study of the Effect of Adding Starch-Modified Natural Rubber Hybrid to the Vulcanization of Sorghum Fibers-Filled Natural Rubber. Polymers.

[34] Xu C., Chen Y., Zheng Z., Liu Y., Cao S., Xu Y. (2020). Mussel-Inspired Biocompatible PAADOPA/PAAm Hydrogel Adhesive for Amoxicillin Delivery. Ind. Eng. Chem. Res..

[35] Yan K.-Y., Chen J.-Y., Li X.-Y., Wang Q., Kuang G.-C. (2020). Carboxylic Acid Enriched Porous Organic Polymer as a Platform for Highly Efficient Removal of Methylene Blue from Aqueous Solution. Macromol. Chem. Phys..

[36] Liufu S., Xiao H., Li Y. (2005). Adsorption of Poly(Acrylic Acid) onto the Surface of Titanium Dioxide and the Colloidal Stability of Aqueous Suspension. J. Colloid Interface Sci..

[37] Mofidfar M., Kim E.S., Larkin E.L., Long L., Jennings W.D., Ahadian S., Ghannoum M.A., Wnek G.E. (2019). Antimicrobial Activity of Silver Containing Crosslinked Poly(Acrylic Acid) Fibers. Micromachines.

[38] Varghese Alex K., Tamil Pavai P., Rugmini R., Shiva Prasad M., Kamakshi K., Sekhar K.C. (2020). Green Synthesized Ag Nanoparticles for Bio-Sensing and Photocatalytic Applications. ACS Omega.

[39] Singh J., Dhaliwal A. (2020). Water retention and controlled release of KCl by using microwave-assisted green synthesis of xanthan gum-cl-poly (acrylic acid)/AgNPs hydrogel nanocomposite. Polym. Bull..

[40] Alim-Al-Razy M., Asik Bayazid G.M., Rahman R.U., Bosu R., Shamma S.S. (2020). Silver nanoparticle synthesis, UV-Vis spectroscopy to find particle size and measure resistance of colloidal solution. J. Phys. Conf. Ser..

[41] Lu Y., Mei Y., Schrinner M., Ballauff M., Möller M.W., Breu J. (2007). In Situ Formation of Ag Nanoparticles in Spherical Polyacrylic Acid Brushes by UV Irradiation. J. Phys. Chem. C.

[42] Abutalib M.M., Rajeh A. (2021). Enhanced structural, electrical, mechanical properties and antibacterial activity of Cs/PEO doped mixed nanoparticles (Ag/TiO_2_) for food packaging applications. Polym. Test..

[43] Zhang X., Chen J., Jiang S., Zhang X., Bi F., Yang Y., Wang Y., Wang Z. (2021). Enhanced photocatalytic degradation of gaseous toluene and liquidus tetracycline by anatase/rutile titanium dioxide with heterophase junction derived from materials of Institut Lavoisier-125(Ti): Degradation pathway and mechanism studies. J. Colloid Interface Sci..

[44] Szente L., Puskás I., Sohajda T., Varga E., Vass P., Nagy Z.K., Farkas A., Várnai B., Béni S., Hazai E. (2021). Sulfobutylether-beta-cyclodextrin-enabled antiviral remdesivir: Characterization of electrospun- and lyophilized formulations. Carbohydr. Polym..

[45] Hou Y., Fang G., Jiang Y., Song H., Zhang Y., Zhao Q. (2019). Emulsion Lyophilization as a Facile Pathway to Fabricate Stretchable Polymer Foams Enabling Multishape Memory Effect and Clip Application. ACS Appl. Mater. Interfaces.

[46] Zhang M., Yang P., Lan G., Liu Y., Cai Q., Xi J. (2020). High crosslinked sodium carboxyl methylstarch-g-poly (acrylic acid-co-acrylamide) resin for heavy metal adsorption: Its characteristics and mechanisms. Environ. Sci. Pollut. Res..

[47] Hou C., Ma K., Jiao T., Xing R., Li K., Zhou J., Zhang L. (2016). Preparation and dye removal capacities of porous silver nanoparticle-containing composite hydrogels via poly (acrylic acid) and silver ions. RSC Adv..

[48] Toader G., Ginghina R.E., Diacon A., Rusen E., Bratu A.E., Podaru A., Rotariu T. (2023). Design and Application of Photocrosslinkable Hydrogel Films for Fast and Efficient Decontamination of Chemical Warfare Agents. ACS Appl. Polym. Mater..

[49] Fan M., Wu L., Hu Y., Qu M., Yang S., Tang P., Pan L., Wang H., Bin Y. (2021). A highly stretchable natural rubber/buckypaper/natural rubber (NR/N-BP/NR) sandwich strain sensor with ultrahigh sensitivity. Adv. Compos. Hybrid Mater..

[50] Hosseini H., Zirakjou A., McClements D.J., Goodarzi V., Chen W.-H. (2022). Removal of methylene blue from wastewater using ternary nanocomposite aerogel systems: Carboxymethyl cellulose grafted by polyacrylic acid and decorated with graphene oxide. J. Hazard. Mater..

[51] Lv Q., Shen Y., Qiu Y., Wu M., Wang L. (2020). Poly(acrylic acid)/poly(acrylamide) hydrogel adsorbent for removing methylene blue. J. Appl. Polym. Sci..

[52] Liu H., Wang Q., Zhang F. (2020). Preparation of Fe_3_O_4_@SiO_2_@ P(AANa-co-AM) Composites and Their Adsorption for Pb(II). ACS Omega.

[53] Xu C., Rangaiah G.P., Zhao X.S. (2014). Photocatalytic Degradation of Methylene Blue by Titanium Dioxide: Experimental and Modeling Study. Ind. Eng. Chem. Res..

[54] Liao C., Li Y., Tjong S.C. (2020). Visible-Light Active Titanium Dioxide Nanomaterials with Bactericidal Properties. Nanomaterials.

[55] Skiba M., Vorobyova V., Pasenko O. (2022). Surface modification of titanium dioxide with silver nanoparticles for application in photocatalysis. Appl. Nanosci..

[56] Chakhtouna H., Benzeid H., Zari N., Qaiss A.E.K., Bouhfid R. (2021). Recent progress on Ag/TiO_2_ photocatalysts: Photocatalytic and bactericidal behaviors. Environ. Sci. Pollut. Res..

[57] Chen Y., Li Q., Li Y., Zhang Q., Huang J., Wu Q., Wang S. (2020). Fabrication of Cellulose Nanocrystal-g-Poly(Acrylic Acid-Co-Acrylamide) Aerogels for Efficient Pb(II) Removal. Polymers.

[58] Ghanei M., Rashidi A., Tayebi H.-A., Yazdanshenas M.E. (2018). Removal of Acid Blue 25 from Aqueous Media by Magnetic-SBA-15/CPAA Super Adsorbent: Adsorption Isotherm, Kinetic, and Thermodynamic Studies. J. Chem. Eng. Data.

[59] Yang J., Wang K., Lv Z., Li W., Luo K., Cao Z. (2021). Facile Preparation and Dye Adsorption Performance of Poly(N-isopropylacrylamide-co-acrylic acid)/Molybdenum Disulfide Composite Hydrogels. ACS Omega.

