# Supplementary material for: The Effect of Silver Nanoparticles/Titanium Dioxide in Poly(acrylic acid-co-acrylamide)-Modified, Deproteinized, Natural Rubber Composites on Dye Removal
Source: Polymers (Basel). 2023 Dec 28;16(1):92. doi: 10.3390/polym16010092 (PMC10780644; doi:10.3390/polym16010092)
Supplement: Supplementary file 1 [file polymers-16-00092-s001.zip › polymers-2772839-supplementary.pdf]

Supplementary Materials: The Effect of Silver Nanoparticles/Titanium Dioxide in Poly(acrylic acid-co-acrylamide)-Modified, Deproteinized, Natural Rubber Composites on Dye Removal

Supharat Inphonlek, Chaiwat Ruksakulpiwat and Yupaporn Ruksakulpiwat

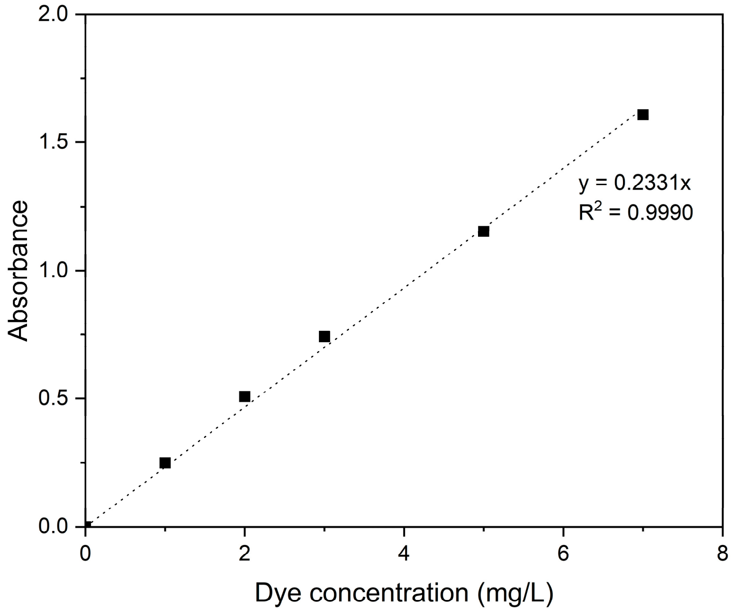

Figure S1. Calibration curve of MB solution.

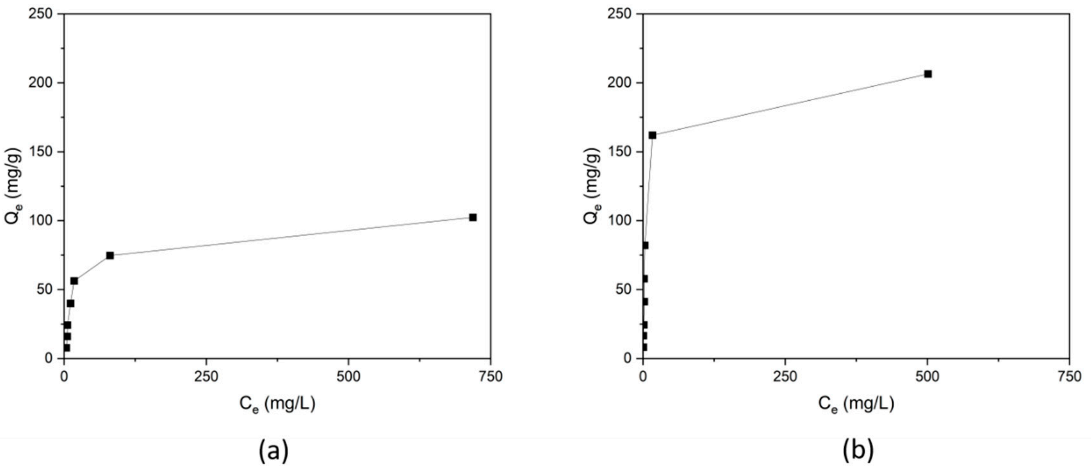

Figure S2. Equilibrium adsorption capacity as a function of equilibrium dye concentration of **(a)** N7, and **(b)** N7/Ag-Ti5.0.
